# Glycinergic dysfunction in a subpopulation of dorsal horn interneurons in a rat model of neuropathic pain

**DOI:** 10.1038/srep37104

**Published:** 2016-11-14

**Authors:** Wendy L. Imlach, Rebecca F. Bhola, Sarasa A. Mohammadi, Macdonald J. Christie

**Affiliations:** 1 The University of Sydney, Discipline of Pharmacology, Sydney, NSW 2006, Australia

## Abstract

The development of neuropathic pain involves persistent changes in signalling within pain pathways. Reduced inhibitory signalling in the spinal cord following nerve-injury has been used to explain sensory signs of neuropathic pain but specific circuits that lose inhibitory input have not been identified. This study shows a specific population of spinal cord interneurons, radial neurons, lose glycinergic inhibitory input in a rat partial sciatic nerve ligation (PNL) model of neuropathic pain. Radial neurons are excitatory neurons located in lamina II of the dorsal horn, and are readily identified by their morphology. The amplitude of electrically-evoked glycinergic inhibitory post-synaptic currents (eIPSCs) was greatly reduced in radial neurons following nerve-injury associated with increased paired-pulse ratio. There was also a reduction in frequency of spontaneous IPSCs (sIPSCs) and miniature IPSCs (mIPSC) in radial neurons without significantly affecting mIPSC amplitude. A subtype selective receptor antagonist and western blots established reversion to expression of the immature glycine receptor subunit GlyRα2 in radial neurons after PNL, consistent with slowed decay times of IPSCs. This study has important implications as it identifies a glycinergic synaptic connection in a specific population of dorsal horn neurons where loss of inhibitory signalling may contribute to signs of neuropathic pain.

Neuropathic pain is a debilitating condition that is caused by a lesion or disease of the somatosensory nervous system and may allow normally innocuous stimuli to generate painful sensations (allodynia) or moderately noxious stimuli to produce excessive pain (hyperalgesia). In contrast to nociceptive pain, neuropathic pain may continue for long periods due to persistent adaptations in central sensory processing mechanisms^1^. These changes in sensory processing may explain why neuropathic pain does not respond well to therapeutics currently used to treat nociceptive and inflammatory pain^2^,^3^.

The spinal cord dorsal horn is the first relay in the central nervous system where nociceptive and innocuous signals are integrated before transmission to the brain. The superficial region of the dorsal horn is innervated by nociceptive fibres (laminae I-II), while the low-threshold mechanoreceptors terminate in deeper laminae (II_i_-VI)^4^. The majority of the cells in laminae I-III of the dorsal horn are interneurons, comprised of both excitatory and inhibitory neurons. Neurons in rodent lamina II have been previously characterised into four groups on the basis of morphology and electrophysiological properties^5^,^6^. These include the inhibitory islet cells with rostrocaudal dendritic trees, inhibitory central cells with shorter rostrocaudal projecting dendrites, excitatory vertical cells with ventrally projecting dendrites and excitatory radial cells that have dendrites that radiate in all directions^7^. In non-pathological states, the excitability of lamina II interneurons is under inhibitory control by glycinergic and GABAergic neurons in lamina III^8^. Several inhibitory components of the pain circuitry in the dorsal horn have been identified that are likely to be involved in allodynia. Coull *et al*.^9^ identified a novel mechanism of disinhibition in lamina I of the dorsal horn following nerve injury, where a reduction in the expression of the potassium-chloride transporter KCC2 results in a shift in the anion gradient leading to synaptic currents becoming less inhibitory and possibly even excitatory. Torsney and MacDermott^10^ identified a polysynaptic pathway in rats that provides a link between Aβ-fibres pathways with lamina I NK1 expressing neurons that are likely to be involved in transmission of noxious information to higher CNS regions. This circuit is usually under strong inhibitory control by glycine and GABA, whereby Aβ-fibres are not able to activate putative nociceptive (NK1 receptor positive) lamina I neurons, but in the presence of bicuculline and strychnine these fibres are able to activate projection neurons in lamina I. However, dysfunction of this circuit in neuropathic pain models was not examined in this study by Torsney and MacDermott^10^. In a later study, Lu *et al*.^11^ built on these findings, showing a convergence of glycinergic inhibitory and excitatory Aβ-fibres inputs onto PCK-γ interneurons in lamina II that form a feed-forward inhibitory circuit which prevents Aβ input from activating nociceptive pathways in rats. This study directly identified a normally silent, excitatory link between the mechanoreceptive and nociceptive pathways in the dorsal horn, which is activated following peripheral nerve-injury or glycine receptor antagonism.

Studies using rat models have shown that loss of inhibitory GABAergic and glycinergic neurotransmission, or disinhibition, accompanies peripheral nerve-injury^12^,^13^,^14^,^15^. This was proposed to be due to apoptotic loss of inhibitory neurons, however there are many discrepancies between studies investigating inhibitory neuron death, which is not thought to be necessary for pain development (reviewed in ref. 4). Regardless of mechanism, loss of inhibition supports the theory that there is a connection linking nociceptive and innocuous circuits, which is under constant inhibitory control^16^.

In order to identify specific components of the pathway that are affected by disinhibition, the present study used patch clamp recording *in vitro* to examine inhibitory neurotransmission onto specific cell types in lamina II after development of tactile allodynia in a rat partial sciatic nerve ligation (PNL) model of neuropathic pain. Profound loss of glycinergic, but not GABAergic, synaptic activity was restricted to one type of cell, the radial neuron. These neurons may contribute to the pain phenotype in neuropathic pain states.

## Results

### Glycinergic signalling is reduced in radial neurons in the spinal cord dorsal horn in nerve-injured animals

**Figure 1:**
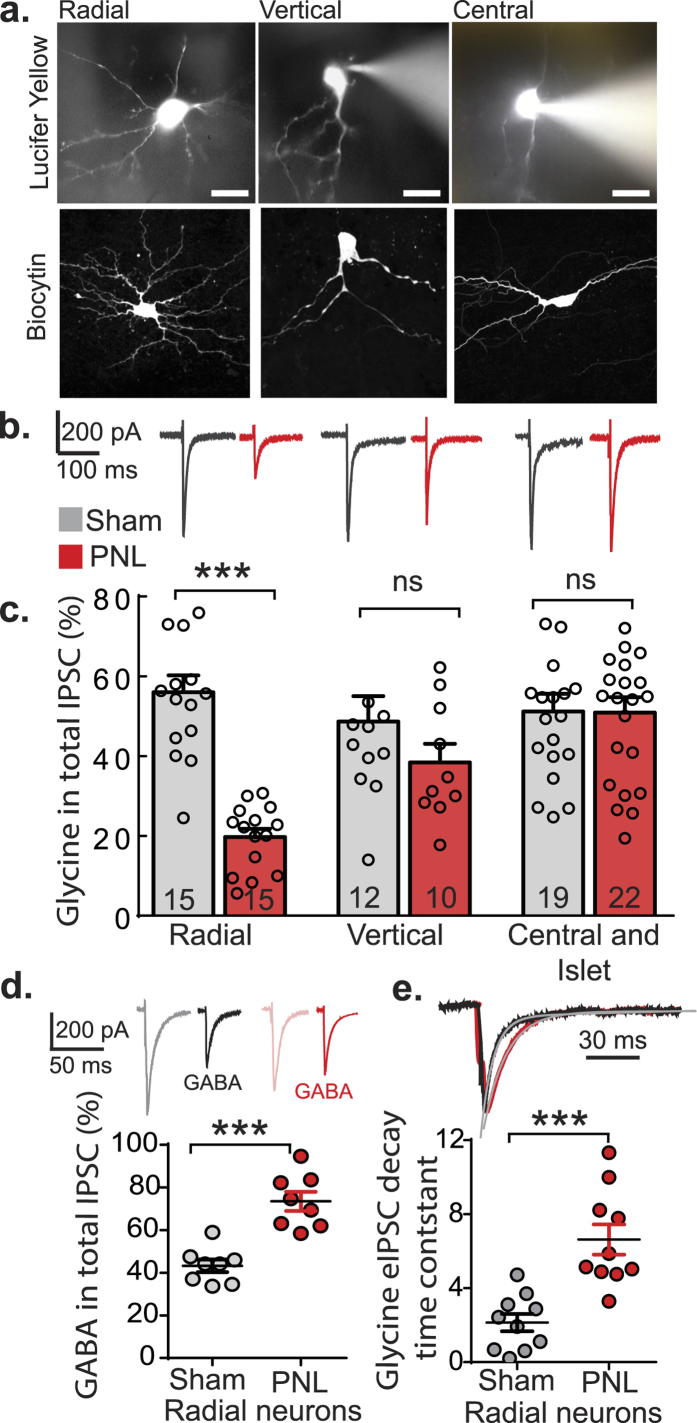
Glycinergic neurotransmission is reduced in radial neurons of superficial dorsal horn after PNL. (**a**) Neurons were classified by their morphology. Micrographs show representative examples of lucifer yellow filled neurons immediately following electrophysiological recording (top row) and biocytin filled neurons imaged by confocal microscopy (bottom row). All cells are shown in the parasagittal plane and the scale bar = 10 μm. (**b**) Examples of glycine-mediated eIPSC traces from control (sham, grey) and nerve-injured (PNL, red) spinal cord neurons. (**c**) Normalized glycinergic eIPSC values as a percentage of the total eIPSC for the neuron types (***p < 0.001, two way ANOVA, Sidak *post-hoc* multiple comparisons test). (**d**) Representative traces of total eIPSCs and GABA eIPSCs, respectively, from radial neurons of sham (grey, black) and PNL (pink, red) animals. Normalized GABA eIPSC values as a percentage of the total eIPSC from radial neurons of control and nerve injured animals. ***p < 0.001 (unpaired *t* test). (**e**) Representative traces from radial cells of control (black) and nerve-injured (red) animals with amplitudes normalized to the first pulse and fitted decay exponential shown in grey. Comparison of glycinergic eIPSC decay time constant from control and nerve injured groups (n =10 for each group) ***p < 0.001 (unpaired *t* tests).

Evoked IPSCs (mixed GABA and glycinergic currents) in parasagittal spinal cord slices were evoked by placing stimulating electrodes in deeper laminae (lamina III or IV) and were measured in lamina II neurons by whole-cell voltage clamp in the presence of CNQX (10 μM) and AP5 (100 μM). The glycinergic component of the eIPSC was then recorded by inhibiting GABA_A_ receptors with addition of picrotoxin (80 μM). Cells were grouped into four morphological categories based on previous descriptions, which included radial, vertical, central and islet^5^,^6^. Morphological criteria for islet and central cells include rostrocaudal dendrites that are long or medium respectively, vertical cells had ventrally projecting dendrites and radial cells had dendrites in all directions. Morphology was determined using lucifer yellow *in situ* in the first instance and some slices were stained *post hoc* for biocytin to confirm morphology (Fig. 1a). Due to similarities in central and islet cells identification with lucifer yellow characterization under the electrophysiology microscope, these two interneuron types were grouped together for analyses. When neurons were classified into these three morphological groups there was 100% concordance between lucifer yellow filled and biocytin stained identification (n = 20 neurons).

Evoked IPSCs for each morphological type from both control (sham) and nerve-injured (PNL) animals were compared to determine the glycinergic contribution. The glycinergic contribution of the eIPSC was determined by measuring the proportion of the eIPSC that remained when the GABA_A_R antagonist, picrotoxin (80 μM) was applied. One subset of neurons, with radial morphology, showed a reduction of 64.8 ± 8.4% in the amplitude of the glycinergic component of the total IPSC following nerve-injury (*p* =< 0.001, two-way ANOVA, Sidak *post-hoc* multiple comparisons test, n = 15 per group, Fig. 1a–c). In contrast, for vertical and central/islet cells there was no significant difference in amplitudes of glycinergic components of the total eIPSC between control and nerve-injured animals (*p* = 0.43 for vertical, n = 12 sham, n = 10 PNL, *p* > 0.99 for central/islet, n = 19 sham, n = 22 PNL, two-way ANOVA, Sidak *post-hoc* multiple comparisons test) (Fig. 1a–c).

To determine whether the reduction in the proportion of the eIPSC sensitive to picrotoxin reflects loss of glycinergic neurotransmission onto radial cells or, alternatively, changes in GlyR or GABA_A_R subunit composition that could affect the pharmacology of picrotoxin^17^,^18^, the sensitivity of the eIPSC to strychnine was determined (Fig. 1d). In the presence of strychnine (0.5 μM), amplitudes of GABAergic eIPSCs were similar to that predicted from the residual amplitude in the presence of picrotoxin, i.e., 56.0 ± 4.2% of the eIPSC was insensitive to picrotoxin (presumably the residual glycinergic component, n = 15) versus a 56.8 ± 2.9% inhibition of the IPSC by strychnine (the direct glycinergic component, n = 8) in sham (*p* = 0.9, unpaired *t* test). After PNL, 19.7 ± 2.1% of the eIPSC was insensitive to picrotoxin versus a 26.5 ± 4.5% inhibition of the IPSC by strychnine (n = 15 and 8 respectively, *p* = 0.1, unpaired *t* test). The GABAergic component of the eIPSC in sham and PNL animals was significantly different (*p* =< 0.001, unpaired *t* test), Fig 1d). Note that this shows a change in the glycinergic component of the total eIPSC, rather than a change in GABAergic signalling. This confirms the loss of glycinergic contribution to the eIPSC in radial neurons of injured animals. The GABAergic components of the eIPSC from central and islet cells were not significantly different in sham and PNL animals (44.0 ± 8.4% of the eIPSC in sham compared to of 54.9 ± 9.8% in injured were insensitive to strychnine, n = 4 and 5 respectively, *p* = 0.44). There was also no difference observed in vertical cells (56.0 ± 4.2% of the eIPSC in sham compared to of 54.2 ± 7.6% in injured were insensitive to strychnine, n = 4 for each group, *p* = 0.85).

When the kinetics of radial neuron glycinergic components of the eIPSCs were compared from control and nerve-injured animals, an increase of 310.0 ± 38.2% in the decay time constant (*p* = 0.0002, unpaired *t* test, Fig. 1e) was observed, which suggests there could be post-synaptic adaptations to glycinergic signalling on the radial neurons, e.g. changes in GlyR subunit composition to subunits, such as GlyRα2 that have slower decay constants^19^. There was no significant difference in eIPSC rise time in control and nerve-injured groups (0.92 ± 0.32 ms and 1.16 ± 0.41 ms respectively, *p* = 0.20, unpaired *t* test). There were no significant differences in decay time constant of the glycinergic components of eIPSCs following nerve injury in central/islet cells (τ = 6.2 ± 0.8 for sham and τ = 6.4 ± 1.0 for nerve-injured, n = 10 and 14 respectively, *p* = 0.8), or in vertical cells (τ = 2.7 ± 0.7 for sham and τ = 2.4 ± 1.1 for nerve-injured, n = 8 and 9 respectively, *p* = 0.8).

### GABA_A_ antagonists bicuculline and picrotoxin have the same effect on eIPSC amplitude and kinetics

**Figure 2:**
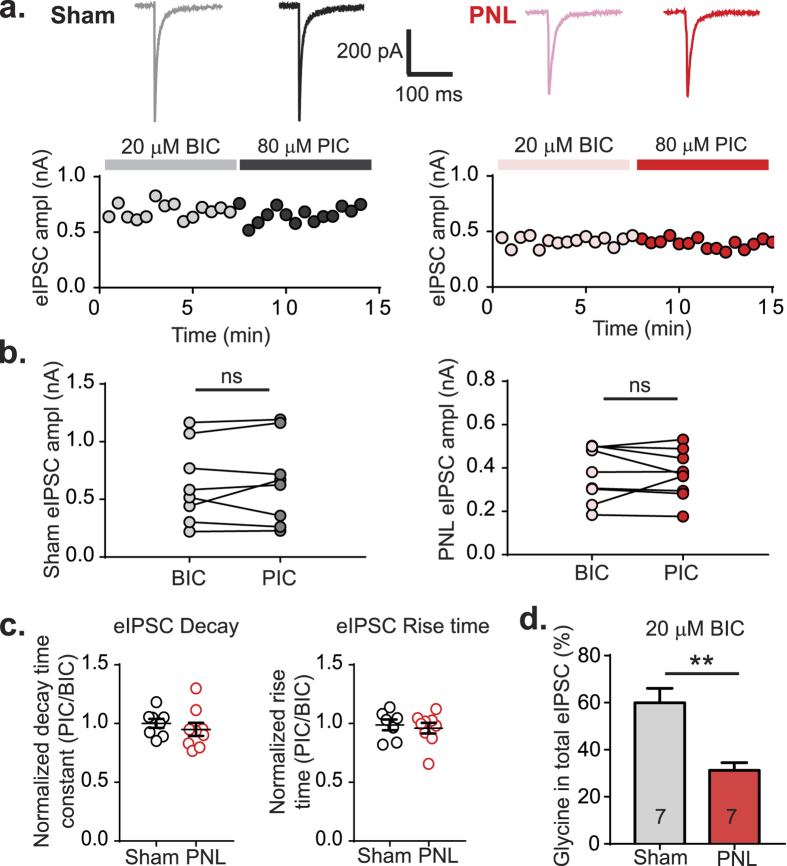
Bicuculline and picrotoxin have similar inhibitory effects on radial neuron eIPSCs. (**a**) Representative traces from a radial neuron superfused with 20 μM bicuculline (BIC) followed by 80 μM picrotoxin (PIC) from sham (grey, black) or PNL (pink, red) with plots showing eIPSC amplitude over time. (**b**) Amplitude of eIPSCs in the presence of bicuculline followed by picrotoxin for sham (grey, n = 8) and PNL (pink/red, n = 9) radial neurons. (**c**) Decay time constant of radial neuron eIPSCs following picrotoxin treatment normalized to the time constant following bicuculline treatment for sham (n = 8) and PNL (n = 9). Rise time of radial neuron eIPSCs following picrotoxin treatment normalized to the rise time following bicuculline treatment for sham (n = 8) and PNL (n = 9). (**d**). Normalized glycinergic eIPSC values (using bicuculline) as a percentage of the total eIPSC (n = 7 for each group). (*p < 0.05, paired *t* test).

In this study picrotoxin was used as a GABA_A_ receptor blocker, however it has been shown that picrotoxin also blocks homomeric glycine receptors, but with very low affinity at the synaptic heteromeric receptors^17^,^18^. A reduction in glycinergic transmission was also observed when the reverse experiment was performed, where strychnine was applied to determine the amount of GABA in the total eIPSC (Fig. 1). However, to further rule out nerve-injury induced expression of homomeric glycine receptors, the effects of competitive GABA_A_ antagonist bicuculline with picrotoxin on eIPSC amplitude and kinetics were compared in the same neurons from dorsal horn of either control or nerve injured animals (Fig. 2). Results showed no significant difference in current size in neurons from control animals using 20 μM bicuculline 0.63 ± 0.12 nA, followed by picrotoxin 0.65 ± 0.13 nA (*p* = 0.67, n = 8, paired *t* test) and in nerve-injured animals using bicuculline 0.38 ± 0.04 nA and picrotoxin 0.37 ± 0.04 nA (*p* = 0.81, n = 9, paired *t* test) (Fig. 2a,b). These results show that both GABA_A_ antagonists have similar effects on eIPSCs in the dorsal horn. In further support of the conclusions that homomeric receptors are not involved post-nerve-injury, rise times and decay time constants of the glycinergic eIPSCs were compared. Since homomeric receptors lack the beta subunit that is required for interaction with gephyrin^20^, they are not clustered at the synapse but are found at extrasynaptic sites. Receptors located more distally from the release sites are likely to have increased rise time as well as increased decay time constants. When eIPSC decay time constants of bicuculline and picrotoxin treated radial neurons were compared, no significant differences were seen for sham (*p* = 0.19, paired t test, n = 8) or nerve-injured (p = 0.28, paired *t* test, n = 9) groups, shown as normalized data in Fig. 2c. Rise times were also not different for each inhibitor within sham (*p* = 0.89, paired *t* test, n = 7) or nerve-injured (*p* = 0.50, paired *t* test, n = 9) groups, also shown as normalized data in Fig. 2c. Furthermore, when rise times of bicuculline treated sham and nerve-injured eIPSCs were compared, there was no significant difference in (*p* = 0.47, unpaired *t* test), which suggests that the synaptic localization of receptors is unchanged following nerve injury. Lastly, the proportion of glycine in the total eIPSC in radial cells from control and nerve-injured animals in experiments using bicuculline (Fig. 2d) were significantly different (*p* = 0.001, unpaired *t* test), similar to results using picrotoxin (Fig. 1c).

### Deficits in glycinergic neurotransmission in radial neurons are associated with presynaptic adaptations

**Figure 3:**
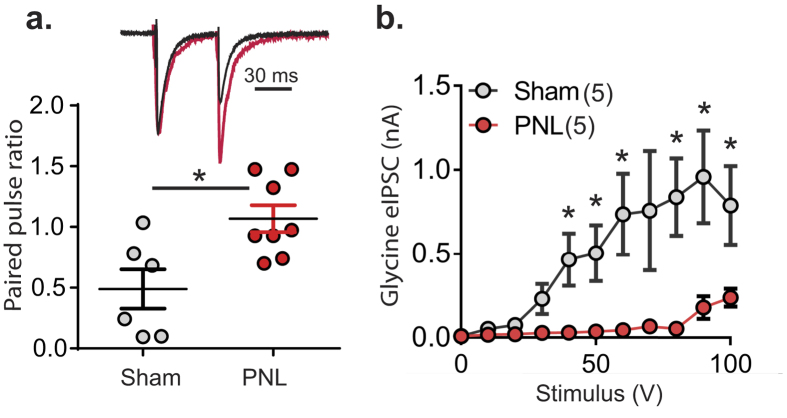
Glycinergic paired-pulse ratio (PPR) increases in radial neurons and more stimulus is required to elicit a response after PNL. (**a**) Representative traces of glycinergic eIPSCs from radial neurons of control (sham, black) and nerve-injured (PNL, red) animals with amplitudes normalized to the first pulse. Scatter dot plot showing data for PPR from control (n = 6) and nerve-injured (n = 8) groups (*p < 0.05, unpaired *t* test). (**b**) Input-output curve showing reduced glycinergic transmission in radial cells from nerve-injured spinal cord at all voltages (*p < 0.05, unpaired *t* test at each voltage value).

To test whether the loss of glycinergic transmission following nerve injury was due to a change in pre-synaptic release probability, paired-pulse ratios of glycinergic eIPSCs were compared between the two groups. PNL increased the paired pulse ratio by 218.0 ± 38.6% (*p* = 0.01, unpaired *t* test, Fig. 3a). This suggests that the probability of glycine release is reduced following nerve injury due to a presynaptic mechanism.

To test the possibility that the loss of glycinergic neurotransmission onto radial neurons might be due to a change in electrical threshold for excitation, a range of stimulus intensities were used to elicit postsynaptic responses in radial neurons from control and nerve-injured animals. Deficits were apparent at all voltages and there were significant differences in response when the voltage exceeded 40 V (Fig. 3b), ie., larger stimuli (90–100 V) were required to elicit glycinergic eIPSCs in radial neurons in the nerve-injured group and the amplitude was always less than in sham controls.

### Spontaneous glycinergic IPSC frequency decreases in radial cells following nerve injury

**Figure 4:**
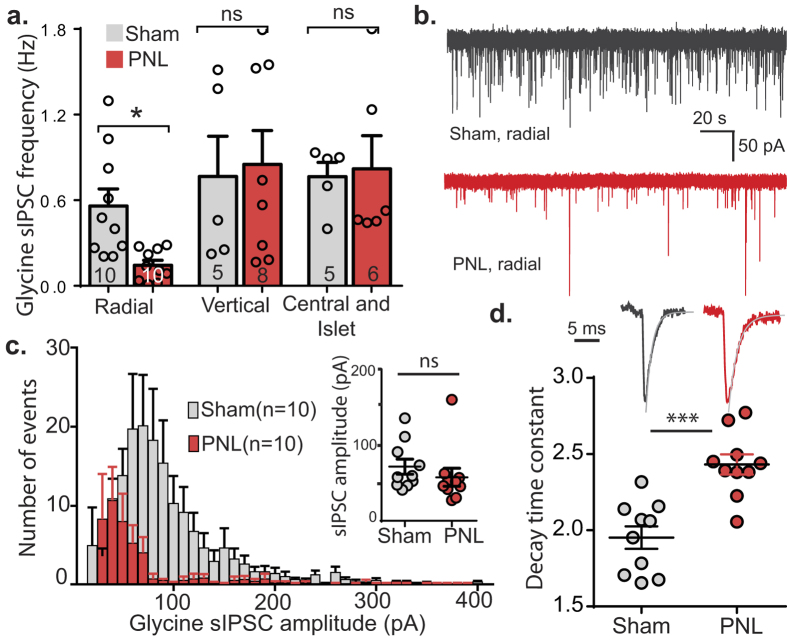
PNL reduces sIPSC frequency and increases decay time in radial neurons. (**a**) Glycinergic sIPSC frequency of radial, vertical and central/islet cells from dorsal horn of control and nerve-injured animals (**p < 0.01, unpaired *t* tests). (**b**) Representative traces of glycinergic sIPSCs from control and nerve-injured radial neurons over a three minute period. (**c**) Amplitude histograms showing distribution of glycinergic sIPSC amplitudes over a 5 minute period for radial neurons from control and nerve-injured animals (n = 10 per group). Mean amplitudes for each cell are shown in the inset. (**d**) Representative traces of sIPSC events (average of 10 events) from control and nerve injured animals with fitted exponential shown in grey and a plot of decay time constants for both treatment groups (n = 10 per group). ***p < 0.001 (unpaired *t* tests).

A 73.9 ± 6.23% decrease in glycinergic spontaneous IPSC (sIPSC) frequency was found in radial cells following nerve-injury (*p* = 0.0492, two-way ANOVA, Fisher’s LSD *post-hoc* multiple comparisons test, Fig. 4a,b), consistent with the decrease in release probability suggested by the paired-pulse ratio experiments. Again, there was no significant difference in sIPSC frequency in vertical and central/islet cells between treatment groups (*p* = 0.93, *p* = 0.83 respectively, two-way ANOVA, Fisher’s LSD *post-hoc* multiple comparisons test, Fig. 4a). From the amplitude histogram (Fig. 4c), the mean amplitude of glycinergic sIPSCs in radial neurons appears to be larger for control animals, with a peak number of events between 60–70 pA, compared to the nerve-injured group which had maximal number of events between 30–40 pA (Fig. 4c). Due to large variation in amplitudes between cells in these two groups, there was no significant difference when mean amplitudes for each group were compared, with a mean of 71.9 ± 9.9 pA for controls and 57.8 ± 11.9 for nerve-injured (*p* = 0.38, Fig. 4c inset). Similar to eIPSCs, an increase in decay time constant of 124.6 ± 3.4% was observed in glycinergic sIPSCs from radial cells following nerve-injury (*p* = 0.0001, Fig. 4d). There was no significant difference in rise time (0.5 ± 0.1 ms for controls, 0.6 ± 0.1 ms for nerve-injured, *p* = 0.16, unpaired *t* test).

### Miniature glycinergic IPSC frequency decreases in radial cells following nerve injury

**Figure 5:**
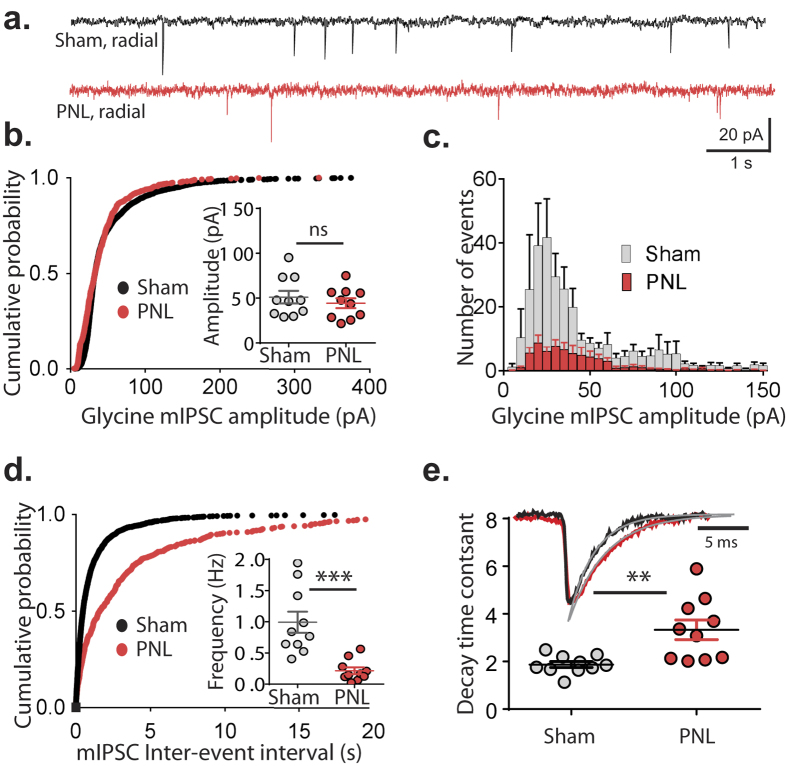
Frequency of mIPSCs in radial neurons are reduced after PNL. (**a**) Representative traces of glycinergic mIPSCs from radial neurons of control (black) and nerve-injured (red) animals over a 10 second period. (**b**) Cumulative probability distribution of glycinergic mIPSC amplitudes of all recordings (n = 10 for each group), with scatter dot plot showing average mIPSC amplitude from each recording (inset). (**c**) Histogram showing distribution of mIPSC amplitude over a 5 minute period for control and nerve injured groups (n = 10 per group). (**d**) Cumulative probability distribution of glycinergic mIPSC inter-event intervals of all recordings (n = 10 for each group), with scatter dot plot showing average mISPC frequency of each recording (inset). (**e**) Representative traces of single mIPSC events from control and nerve-injured animals with fitted exponential shown in grey and a scatter dot plot of decay time constants for both treatment groups (n = 10 per group). **p < 0.01; ***p < 0.001 (unpaired *t* tests).

The large reduction in glycinergic sIPSC frequency onto radial cells from injured animals, together with the increase paired-pulse ratio, is consistent with reduced glycine release probability. However, changed decay times of eIPSCs and sIPSCs suggest post-synaptic adaptations might also contribute. Miniature IPSCs (mIPSCs) were thus recorded to obtain further information on release probability and quantal amplitude. Recordings in the presence of TTX showed no change in mIPSC amplitude in radial neurons between control (51.1 ± 7.1 pA) and PNL (44.3 ± 5.5 pA) animals (*p* = 0.46, Fig. 5a,b). However, the number of events over a 5 minute period was greatly reduced in the PNL group, with a 78.4 ± 5.0% decrease in mIPSC frequency (*p* = 0.0004, unpaired *t* test Fig. 5a,c,d). The effect of nerve-injury on decay time of glycinergic mIPSCs was similar to that seen in eISPCs and sIPSCs, with a 177.9 ± 22.0% increase in decay time constant in the nerve-injured group (*p* = 0.003, unpaired *t* test Fig. 5e). There was no significant difference in mIPSC rise time in control and nerve-injured groups (0.63 ± 0.16 ms and 0.61 ± 0.11 ms respectively, *p* = 0.83, unpaired *t* test).

### Loss of glycinergic input to radial cells is not due to activation of EP2 receptors by prostaglandins

**Figure 6:**
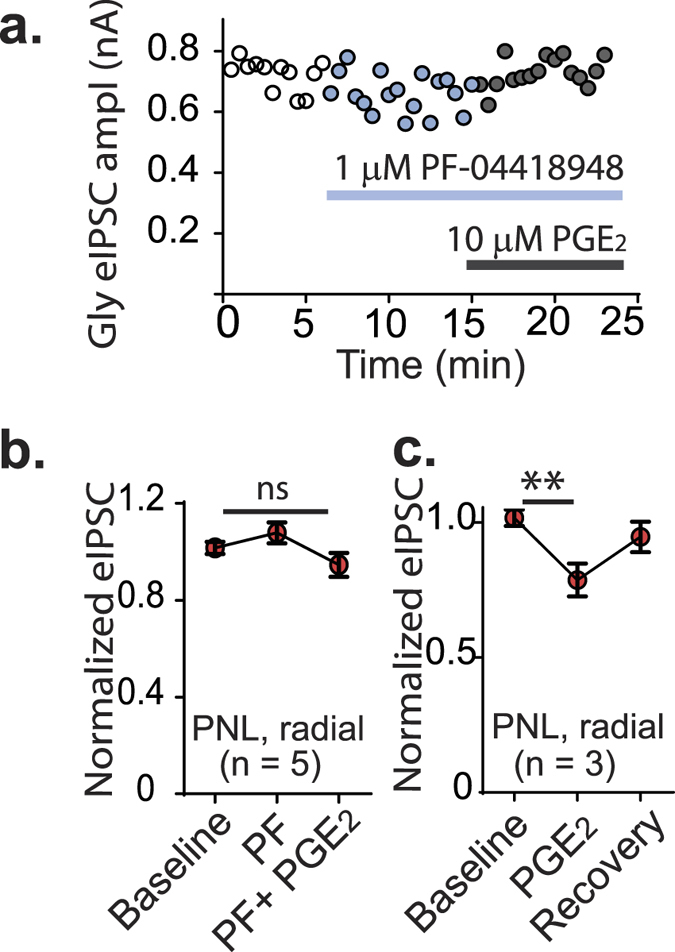
Reduction in eIPSC is not due to EP receptor activation by PGE_2_. (**a**) Time plot showing glycinergic eIPSC amplitude in the presence of the EP receptor antagonist PF-04418948, followed by PGE_2_. (**b**) Histograms show normalized glycinergic eIPSC in response to PF-04418948 and (**c**) in response to PGE_2_ in the absence of the EP antagonist. *p < 0.05 (paired *t* tests).

In chronic inflammatory pain, release of prostaglandin E2 (PGE_2_) activates EP receptors, which in turn reduces glycinergic signalling through GlyRα3^21^. To determine whether glycine receptors on radial neurons were inhibited by release of PGE_2_ following nerve injury, eIPSCs in radial neurons were measured in the presence of an EP receptor antagonist, PF-04418948, which blocks activation by PGE_2_. No significant difference was found in glycinergic eIPSC amplitude from radial neurons when PF-04418948 (1 μM) was superfused (*p* = 0.564, two way ANOVA with Tukey’s multiple comparisons test, Fig. 6a,b). To confirm that the concentration of the EP antagonist was sufficient to block EP receptors, 10 μM PGE_2_ was applied to slices in the presence of the antagonist which showed no change over the three conditions (*p* = 0.14, two way ANOVA, Fig. 6a,b). In the absence of the EP antagonist, 10 μM PGE_2_ reduced glycinergic eIPSCs by 24.0 ± 0.1% (*p* = 0.008, two way ANOVA with Tukey’s multiple comparisons test, Fig. 6c).

### The GlyR α2 subunit is expressed following nerve injury

**Figure 7:**
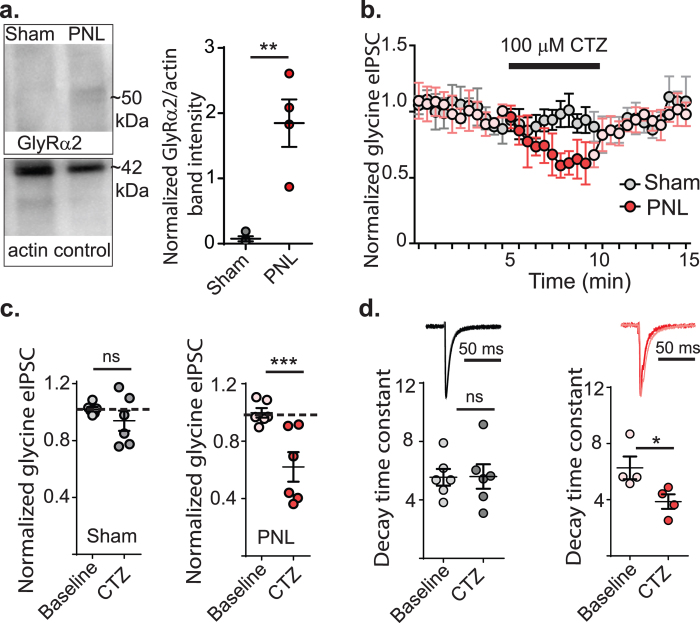
Cyclothiazide decreases glycinergic eIPSC amplitude in radial cells from nerve-injured animals. (**a**) Western blot detection of GlyRα2 from membrane proteins from the lumbar dorsal horn of control and nerve-injured animals. Actin is used as a loading control. Scatter dot plot shows densitometry of GlyRα2 western blot bands normalized to the loading control and then the sham control. **p < 0.01 (unpaired *t* test). Full length blots are presented in Supplementary Figure 1. (**b**) Time plot showing normalized glycinergic eIPSC amplitudes from radial cells of control (red) and nerve injured (grey) animals in response to cylclothiazide (CTZ). Darker filled circles show time points where drug is superfused. Mean taken between 2–4 minutes (baseline) and 8–10 minutes (CTZ), normalized to the first 5 minutes of recording. (**c**) Plots of eIPSC response to CTZ from control and nerve-injured tissue. ***p < 0.001 (two way ANOVA with Tukey multiple comparisons test). (**d**) Representative trace and dot plot showing decrease in decay time following application of CTZ in radial cells from nerve-injured animals.**p < 0.01 (paired *t* tests).

The increase in decay time constants for evoked, spontaneous and miniature IPSCs after PNL without any effect on rise time kinetics might be explained by increased expression of the glycine receptor α2 subunit, which has slower decay kinetics than the other glycine receptor subunits when expressed as either homomeric or heteromeric channels^19^. This subunit is important developmentally and expressed embryonically but expression declines in rat spinal cord within the first two postnatal days, so its presence would not be expected in the adult^22^. To test this, a western blot was performed on membrane proteins from the dorsal horn and tested for the presence of GlyRα2 using an antibody specific for the C-terminus of this subunit (Fig. 7a). A faint band was detected in PNL tissue by western blot that was of the expected size for a GlyRα2, which was not detected in dorsal horn tissue from sham animals (n = 4 for each group). The intensity of the immunoreactive band in PNL tissue was quantified by background subtracted optical densitometry and normalized to the actin loading control. Densitometry values for PNL tissue was found to be 25-fold darker compared to the same MW region of the sham control blot (0.075 ± 0.038 in sham, 1.85 ± 0.36 in PNL; *p* = 0.0028, unpaired *t* test) (Fig. 7a).

If GlyRs that include α2 subunits are expressed in synapses of radial neurons after nerve-injury then then eIPSCs should be susceptible to the selective GlyRα2 antagonist, cyclothiazide^23^. Clyclothiazide (100 μM) had no effect on eIPSC amplitude in radial neurons of control animals (*p* = 0.67, two way ANOVA, Tukey *post-hoc* multiple comparisons test, n = 6, Fig. 7b,c), but produced a significant decrease after nerve-injury (37.9 ± 10.3%, *p* = 0.0003, two way ANOVA, Tukey *post-hoc* multiple comparisons test, n = 6, Fig. 7b,c), which suggests that GlyRα2 containing GlyRs are expressed only after nerve-injury. Moreover, the decay time constant was reduced by 38.3 ± 8.1% in radial cells in the presence of cyclothiazide from nerve-injured animals (*p* = 0.047, Fig. 7d) but there was no effect in controls (*p* = 0.96, Fig. 7d). Non-radial neurons (vertical, islet and central; n = 4) from PNL animals showed no change in current amplitude in the presence of cyclothiazide. Together with the prolonged IPSC decay kinetics and immunochemistry after PNL, this supports the interpretation that a component of the eIPSC in radial cells is mediated through receptors containing GlyRα2 subunits after nerve-injury.

## Discussion

The importance of glycinergic transmission in normal pain and touch circuitry has been known since Yaksh^14^ reported allodynia in rats after administering strychnine to the spinal cord to block glycinergic transmission. Since then, nociceptive circuitry has been further deciphered to reveal neurons and synaptic contacts that may contribute to pathological pain. Much of the work in the dorsal horn has focussed on PKC-γ expressing interneurons, which include a number of neuronal subtypes throughout lamina II. Malmberg *et al*.^24^ suggested that PKC-γ expressing interneurons play a role in neuropathic pain because PKC-γ knock-out mice were resistant to neuropathic pain development. More recently, Miraucourt *et al*.^25^ reported that allodynia in a neuropathic pain model could be prevented with selective PKC-γ inhibition. Lu *et al*.^11^ showed that PKC-γ expressing neurons formed part of a feed-forward inhibitory circuit that prevents Aβ input from activating nociceptive pathways in rats. Recently, Alba-Delgado *et al*.^26^ found that the PKC-γ-positive interneurons in lamina II of the medullary dorsal horn could be classified into two morphologically and functionally different subpopulations: central and radial PKC-γ-positive interneurons, with PKC-γ-negative interneurons mostly vertical and central. This suggests that the radial cells in the current study are likely to be in the PKC-γ-positive category.

Zeilhofer *et al*.^27^ used enhanced green fluorescent protein driven by the glycine transporter 2 (GlyT2) promoter to show that glycinergic neurons are expressed predominantly in lamina III and V of the dorsal horn (illustrated in Zeilhofer *et al*.^28^). Todd *et al*.^29^ showed colocalization of GABA_A_ and glycine receptors at the same synapse of dorsal horn neurons. The locations of inhibitory neurons in the rat dorsal horn have previously been mapped by Takazawa and MacDermott^30^, who showed a gradient of inhibitory input that is predominantly glycinergic in the inner lamina II region, to mostly GABAergic in outer lamina II through to lamina I. Consistent with this, glycinergic eIPSCs could only be evoked reliably in the present study when stimulating electrodes were place in lamina III (see Methods). Since fibres transmitting low threshold innocuous input terminate in lamina III and inner lamina II, it was previously concluded that glycinergic inhibition is likely to be important in this region. Dorsal horn circuits were also investigated by Torsney and MacDermott^10^ who found a pathway that links Aβ-fibres with lamina I projection neurons. More recently, Peirs *et al*.^31^ identified VGLUT3 expressing neurons in the deep dorsal horn of mice that appear to be at the interface between touch and pain circuits. These neurons receive innocuous input and transmit the signals dorsally causing mechanical allodynia. They also showed that different microcircuits are recruited in different pain types with mouse neuropathic models signalling through a PKC-γ mediated circuit and mouse inflammatory models through cells that express calretinin. Data from the present study provides further information on this spinal pain circuitry and identifies radial neurons as a potentially important component of the circuits affected by disinhibition.

### Radial neurons lose glycinergic signals in neuropathic pain, yet GABA signalling remains the same

In the present study, the results for eIPSCs sIPSCs and mIPSCs all showed that PNL greatly reduces the activity of glycinergic synapses, resulting in a preferential loss of glycinergic transmission to lamina II radial neurons, without affecting vertical or central/islet cells. This suggests that the basal glycinergic inhibitory activity onto radial neurons is severely compromised following the development of neuropathic pain. The causes for this specific deficit are not yet known, but the decrease in paired pulse ratios suggests that presynaptic release probability mechanisms contribute, at least in part. This may be due to loss of terminals or a reduction in the probability of release from terminals. Since the amplitude of mIPSCs did not change, the data suggests that that the glycine receptor on the postsynapse is still present and functional, but the probability of release from the presynapse is reduced. Experiments comparing the effects of bicuculline and picrotoxin on glycinergic eIPSCs suggest that the synaptic localization of receptors is unlikely to be changed following nerve-injury. Amplitude of the sIPSC events from the sham group were twice the amplitude of the PNL group which, in the context of no change in mIPSC amplitude, suggests there is more summation or synchronous release in the control group.

A mechanism for loss of inhibitory neurotransmission in neuropathic pain has been previously described by Coull *et al*.^9^, who found that nerve-injury causes a depolarizing shift in the chloride equilibrium of lamina I cells in the dorsal horn. This disruption to anion homeostasis results from reduced expression of the potassium-chloride exporter KCC2, which results in both GABAergic and glycinergic currents becoming less inhibitory and possibly even excitatory. However, if this switch is occurring in lamina II, this would be expected to affect chloride currents resulting from both GABA and glycine signalling. Moreover, the present experimental setup would be very unlikely to detect such changes because the postsynaptic cells are chloride loaded using a CsCl internal solution in patch pipettes.

In chronic inflammatory pain, glycinergic signalling through GlyRα3 is reduced in response EP receptor activation by prostaglandins^21^. To determine whether glycine receptors on radial neurons were inhibited by release of PGE_2_, experiments were performed to measure the influence of EP receptor activation on evoked glycinergic current amplitude. The data showed no change in glycinergic eIPSC amplitude in the presence of the EP receptor antagonist, PF-04418948, which blocks the activity of PGE_2_ at this receptor. This suggests that the reduction in glycinergic signalling in these neurons is not mediated through inhibition of the GlyRα3 following EP receptor activation by PGE_2_. This is consistent with the report by Hösl *et al*.^32^ that shows glycinergic neurotransmission by EP2 receptor activation does not contribute to pain in nerve-injury models.

Surprisingly, the dramatic reduction in glycinergic signalling was not seen with GABA signalling at the inhibitory radial synapse (Fig. 1). GABA and glycine are co-released at many inhibitory synapses in the dorsal horn lamina I-II in adult rats, with individual postsynaptic currents (mIPSCs) mediated by either the GABA receptors or glycine receptors^33^. These differences may be due to different regions of inhibitory neurons being affected. It has previously been shown that there is a gradient of inhibitory input in the dorsal horn with more glycinergic inhibition in inner lamina II and increasing GABA input on the dorsal region of lamina II and in lamina I^30^.

### Glycinergic transmission on radial neurons is influenced by postsynaptic changes in nerve injury

The results strongly suggest that nerve-injury induces increased expression in radial neurons of glycine receptors containing GlyRα2 subunits. Glycine receptors are pentameric channels made up of either homomeric assemblies of α subunits or heteromeric assemblies of α and β subunits^34^. These receptors show a restricted distribution in the dorsal horn with both GlyRα1/β and GlyRα3/β forms expressed in the adult spinal cord, the latter being specific to nociceptive neurons^35^. Immature glycine receptors, which are important embryonically for development, assemble as GlyRα2 homomeric channels, but expression declines after embryonic development in the two postnatal days^22^. However, heteromeric channels can assemble from GlyRα2 with beta subunits^19^ and these have slower decay time than receptors composed of the GlyRα1/β or GlyRα3/β subunits. The increase in the synaptic current decay time constant observed in evoked, spontaneous and miniature IPSCs after PNL is therefore consistent with increased contribution of GlyRα2 subunits to IPSCs. The increased sensitivity of the IPSC to cyclothiazide after PNL, which has been shown to selectively inhibit the GlyRα2 subunits without inhibiting GlyRα1 or GlyRα3^23^ supports the induction of GlyRα2 subunits, which was confirmed by Western blots. Levels of GlyRα2 detected in dorsal horn of PNL animals were very low, which might be expected if the expression of this receptor induced by PNL is restricted to radial neurons. Thus GlyRα2 subunits, that are not usually expressed in adult spinal cord^19^, reappear after nerve-injury. It is tempting to speculate that this may be an adaptive response to injury wherein gene expression partly reverts to the early expressed or embryonic expression pattern in pathological conditions.

Although embryonically expressed GlyRα2 containing channels are homomeric, it is more likely that heteromeric GlyRα2 containing channels are responsible for the present results. Homomeric GlyRα2 channels are blocked potently by picrotoxin (IC_50_ = 0.79–4.1 μM in HEK cells depending on splice variant^36^) but not bicuculline. However, the effects of these two antagonists on IPSCs were indistinguishable, strongly suggesting the synaptic effects of picrotoxin did not involve homomeric GlyRα2 channels. Because they do not include a synaptic targeting β subunit, homomeric GlyRα2 channels would be expected to be located at extrasynaptic sites^20^,^37^. If synaptically released glycine were to diffuse to extrasynaptic GlyRs then a delayed or slowed synaptic rise time would be expected. However, no increase in rise time was observed for the picrotoxin sensitive component of eIPSC, sIPSCs or mIPSCs after PNL.

Previous reports suggest that GlyRα2 homomeric receptors are unsuitable for fast synaptic transmission because they activate and deactivate slowly and have low probability of opening in response to the rapid application of brief glycine responses^38^. If a substantial proportion of inhibitory synapses express GlyRα2 homomeric receptors after PNL then it is possible that the low probability of activation of GlyRα2 homomeric receptors during synaptic release of glycine (ibid.) contributes to the reduced probability of glycinergic synaptic activity observed here.

**Figure 8:**
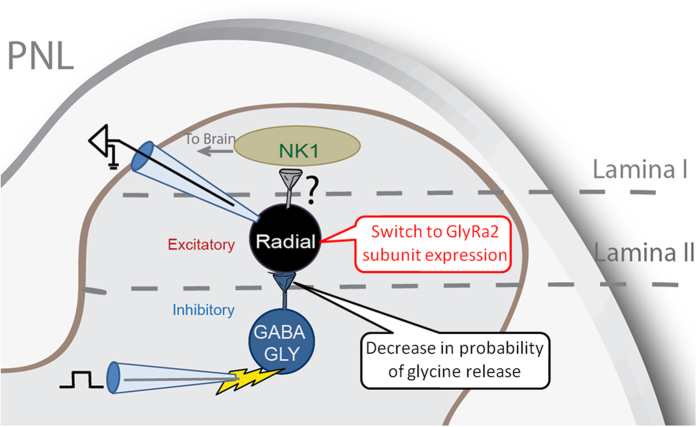
Schematic showing recording configurations used in this study and main findings. Inhibitory neurons in lamina III were stimulated and synaptic currents in lamina II interneurons were recorded. Results show a decrease in the probability of glycine release from glycinergic neurons that synapse with radial neurons, as well as a switch to GlyRα2 subunits in radial neurons.

In conclusion, a population of dorsal horn neurons with impaired glycinergic signalling in neuropathic pain has been identified in this study. In normal states, these neurons receive both GABAergic and glycinergic synaptic input from ventrally positioned inhibitory neurons, but pathological changes result in presynaptic loss of glycinergic signalling, as well as postsynaptic changes in glycine receptor expression (Fig. 8). Although the importance of inhibitory neurotransmission in the spinal cord has been known for some time, to our knowledge, this is the first time a distinct subset of spinal interneurons has been identified that have impaired glycinergic neurotransmission. These neurons are likely to play an important role in the development of allodynia and perhaps other sensory features of chronic neuropathic pain. Further characterization of adaptations to radial neuron function in chronic pain models may lead to novel therapeutic treatments.

## Methods

### Neuropathic pain model

Neuropathic pain was induced in 5–6 week old male Sprague-Dawley rats (n = 75) by performing a partial nerve ligation (PNL) of the left sciatic nerve^39^, as described previously^40^. Briefly, rats were anaesthetized with isoflurane and the sciatic nerve proximal to its trifurcation was surgically exposed and a single suture was tied around one third to one half of the nerve. Rats were assessed for mechanical allodynia two weeks post-PNL surgery using a von Frey assay (see below) and used for electrophysiology experiments between 2.5–4 weeks post-surgery. Sham surgery rats with all other procedures but no nerve ligation were used as controls (n = 68). Rats were housed in a temperature controlled environment 22 ± 2 °C with a 12 hour light/dark cycle. Animals were housed in groups of 3 or 4 and had free access to food and water. All experiments involving animals were approved by the University of Sydney Animal Ethics Committee. Experiments were performed under the guidelines of the Australian code of practice for the care and use of animals for scientific purposes (National Health and Medical Research Council, Australia, 7th Edition). Every precaution was taken to prevent animal suffering during these experiments.

### Mechanical allodynia testing

Mechanical paw withdrawal threshold (PWT) was used to assess mechanical allodynia to confirm the development of neuropathic pain prior to electrophysiology experiments. To measure mechanical PWT, animals were acclimatized to clear Perspex containers with a steel mesh floor several times before the day of experiment, and for around 15 min prior to testing on the day of experiment. A series of von Frey filaments (0.41–15 g) were presented using an up-down paradigm to calculate 50% withdrawal threshold^41^. Mechanical PWT was tested prior to surgery on day 0 and 14 days following surgery. A reduction in von Frey threshold from a pre-surgery baseline of (14.9 ± 0.4 g, n = 58) to below 4 g 14 days after surgery was used as a threshold criterion that neuropathic pain had developed. 73 animals met this threshold with an overall threshold of 0.9 ± 0.1 g (n = 56), i.e. only 2 animals were not used for electrophysiology experiments.

### Preparation of spinal cord slices

Adult male Sprague-Dawley rats (8–10 weeks at the time of slice preparation) (n = 141 PNL rats and sham controls) were anaesthetized with isoflurane, decapitated and the lumbar region of the spinal cord was removed. Parasagittal slices (340 μm thick) of spinal cord were cut on a vibratome (Leica VT 1200 s) in oxygenated ice-cold sucrose-based artificial CSF (sACSF) that contained (mM): 100 sucrose, 63 NaCl, 2.5 KCl, 1.2 NaH_2_PO_4_, 1.2 MgCl_2_, 25 glucose, and 25 NaHCO_3_. Slices were transferred to a submerged chamber containing NMDG-based recovery ACSF (rACSF) for 15 minutes at 34 °C, equilibrated with 95% O_2_ and 5% CO_2_ and composed of (mM): 93 NMDG, 2.5 KCl, 1.2 NaH_2_PO_4_, 30 NaHCO_3_, 20 HEPES, 25 Glucose, 5 Na ascorbate, 2 thiourea, 3 Na pyruvate, 10 MgSO_4_ and 0.5 CaCl_2_, and adjusted to pH 7.4 with HCl. Following the recovery incubation, slices were transferred to normal oxygenated ACSF where they were allowed to recover for 1 hour at 34 °C and then maintained at room temperature prior to transfer to the recording chamber. Normal ACSF had the following composition (mM): 125 NaCl, 2.5 KCl, 1.25 NaH_2_PO_4_, 1.2 MgCl_2_, 2.5 CaCl_2_, 25 glucose, and 11 NaHCO_3_ and was equilibrated with 95% O_2_ and 5% CO_2_.

### Electrophysiology

Slices were transferred to a recording chamber and superfused continuously at 2 ml/min with normal ACSF that had been equilibrated with 95% O_2_ and 5% CO_2_ and maintained at 34 °C with an inline heater and monitored by a thermister in the slice chamber. Dodt-contrast optics was used to identify lamina II neurons in the translucent substantia gelatinosa layer of the superficial dorsal horn. The morphology and position of lamina II cells were identified by lucifer yellow fluorescence *in situ* once recording was completed to facilitate classification of all neurons whether or not they could be recovered *post hoc*. Neurons were also filled with biocytin for *post hoc* identification. A Cs^+^-based internal solution, which should minimise postsynaptic effects, was used to record electrically evoked inhibitory post-synaptic currents (eIPSCs) and contained (mM): 140 CsCl, 10 EGTA, 5 HEPES, 2 CaCl_2_, 2 MgATP, 0.3 NaGTP, 5 QX-314.Cl, 2 Lucifer Yellow CH dipotassium salt and 0.1% biocytin (osmolarity 285–295 mosmol l^−1^). Patch electrodes had resistances between 3 and 5 MΩ. Synaptic currents were measured in whole-cell voltage-clamp (−70 mV, not corrected for a liquid junction potential of 4 mV) from lamina II cells. Bipolar tungsten electrodes placed in the inner laminae (lamina III region) were used to elicit eIPSCs using a stimulus strength sufficient to evoke reliable eIPSCs. In this study, we electrically stimulated neurons ventral to lamina II, in regions that are known to contain glycinergic neurons. Glycinergic IPSCs could not be elicited when from more superficial sites or when the stimulating electrode was moved to more ventral regions of the dorsal horn (deeper in lamina IV-V). For paired pulse experiments, evoked currents were elicited by two consecutive stimuli of identical strength separated by 50 ms. Paired pulse ratio (PPR) was calculated by dividing the second pulse by the first (PSC_2_/PSC_1_). All eIPSCs were recorded in CNQX (10 μM), AP5 (100 μM) as well as either strychnine (0.5 μM) or picrotoxin (80 μM). Spontaneous inhibitory post-synaptic currents (sIPSCs) were recorded for a 5 minute period under the same conditions as eIPSCs but with no stimulation. Miniature spontaneous IPSCs (mIPSCs) were recorded under the same conditions for 5–10 minutes with tetrodotoxin (1 μM) included in the extracellular solution. IPSC decay constants for were determined using the fit exponential tool in Axograph X to fit the current from an average of 10 consecutive IPSCs for each condition. A mono-exponential function was fitted using a Simplex algorithm. Rise times were measured between 10–90% of the current peak using a measure peaks tool in Axograph. At the conclusion of each experiment strychnine (0.5 μM) or picrotoxin (80 μM) were added to the superfusion solution (i.e. strychnine + picrotoxin present) to confirm that recorded currents were glycine- mediated IPSCs or GABA-mediated IPSCs, respectively. Drugs were superfused onto slices at a rate of 2 ml/min in normal oxygenated ACSF at 34 °C.

### Biocytin labelling

At the end of electrophysiological recordings, some slices were fixed in 4% paraformaldehyde in 0.1 M phosphate buffered (PB) that contained (M) 0.019 NaH_2_PO_4_2H_2_O and 0.081 Na_2_HPO_4_, pH 7.4 for 2–4 hours and then washed in 0.1 M PB. Slices were then incubated at 4 °C overnight in 0.3% Triton X-100 in 0.1 M PB. The following day, slices were incubated at room temperature in 5% normal horse serum, 1% bovine serum albumin and 0.3% Triton X-100 in 0.1 M PB for 1 hour. Slices were incubated for 2 hours at room temperature with Cy5-Streptavidin (Sigma) diluted in 1% BSA and 0.3% Triton X-100 in 0.1 M PB to stain biocytin-filled cells. Slices were washed in 0.1 M PB and mounted onto slides, briefly air dried and immersed in Fluoromount-G (ProSciTech). Images of labelled neurons were obtained with a confocal microscope.

### Western blot

Adult male Sprague-Dawley rats (8–10 weeks) (n = 4 PNL rats and 4 sham controls) were anaesthetized with isoflurane, decapitated and the lumbar region of the spinal cord was removed. Horizontal slices (600 μm thick) containing the dorsal horn region of the spinal cord were cut using a vibratome (Leica VT 1200 s) in ice-cold sucrose-based artificial CSF (sACSF) that contained (mM): 100 sucrose, 63 NaCl, 2.5 KCl, 1.2 NaH_2_PO_4_, 1.2 MgCl_2_, 25 glucose, and 25 NaHCO_3_. The injured (left) side (and the left side in sham animals) was removed from the uninjured (right) side using a blade. Tissue isolated from the left side was immediately frozen in liquid nitrogen and stored at −80 °C until required. Thawed tissue was homogenized in lysis buffer containing: 10 mM Tris HCl pH 7.4, 0.32 M sucrose, 5 mM EDTA pH 8, supplemented with 1% protease inhibitor cocktail (Sigma), then mixed with an equal volume of 4% SDS, and sonicated. Membrane fractions were collected by centrifugation at 13,000 *g* (4 °C) for 20 min with three washes in lysis buffer. The remaining pellet was resuspended in RIPA buffer containing: 25 mM TrisCl pH 7.6, 150 mM NaCl, 1% Triton X-100, 1% sodium deoxycholate, 1% SDS, with 1% protease cocktail inhibitor, and incubated for 10 min at room temperature. Protein concentrations were determined using a BCA protein assay (Pierce, Thermo Scientific) and absorbance was measured using a FLUOstar Omega microplate reader (BMG Labtech). Samples containing 30 μg protein were denatured by heating in sample buffer containing SDS and 2-mercaptoethanol and separated by electrophoresis on a 10% SDS-PAGE gel. The protein was then electrotransferred onto Amersham Hybond 0.45 μm PVDF membrane (GE Healthcare) and membranes were blocked in PB containing 2% BSA with 0.1% Tween20. Presence of the GlyRα2 subunit was detected using an antibody against the C-terminus (sc-20133^42^, Santa Cruz) and a species specific biotinylated secondary antibody (Amersham Biosciences) followed by an ExtrAvidin Peroxidase conjugate (Sigma). Actin was used as a loading control and detected using an anti-actin antibody (Sigma) followed by a peroxidise conjugated secondary antibody (Jackson Immunoresearch). Membranes were incubated in Pierce ECL Plus Western blotting substrate (Thermofisher) and bands visualized using a ChemiDoc MP system (Bio-Rad). Western blots were analysed by measuring the intensity of immunoreactive bands by optical densitometry using Bio-Rad Image Lab V 5.2.1 software. Western blot background was measured from area of the gel where there was no band and the density of bands was quantified relative to this region. Density of GlyRα2 bands in PNL lanes (or the equivalent MW region in lanes from sham controls) were normalized to the band density of the actin loading control.

### Drugs and chemicals

Picrotoxin, Bicuculline, CNQX and strychnine were purchased from Sigma Australia. QX-314 was purchased from Alomone Labs, Israel. APV, bicuculline and cyclothiazide were purchased from Tocris Bioscience, UK. PF-04418948 and PGE_2_ were purchased from Sapphire Bioscience, Australia. All other chemicals were purchased from Sigma, Australia unless otherwise stated in the text.

### Data analysis

Pooled values are presented as mean ± SEM, or drug effect normalized to the baseline. Data were analysed using Prism 6 (Version 6.03). Statistical tests between treatment groups were made using a two-tailed unpaired *t* test assuming unequal variance. Comparisons of two treatments in the same group were made using a two-tailed paired *t* test. When multiple comparisons were tested, ANOVA with Sidaks or Fishers LSD *post hoc* test to correct for multiple comparisons were used, unless otherwise stated in figure legends. Normalized concentration-response data were pooled and fitted with a logistic function using Prism 6 software. Significance was set at *P < 0.05, **P < 0.01, ***P < 0.001 levels.

## Additional Information

**How to cite this article**: Imlach, W. L. *et al*. Glycinergic dysfunction in a subpopulation of dorsal horn interneurons in a rat model of neuropathic pain. *Sci. Rep*. **6**, 37104; doi: 10.1038/srep37104 (2016).

**Publisher’s note**: Springer Nature remains neutral with regard to jurisdictional claims in published maps and institutional affiliations.

## Supplementary Material

Supplementary Information
