# Supplementary material for: Glycinergic dysfunction in a subpopulation of dorsal horn interneurons in a rat model of neuropathic pain
Source: Sci Rep. 2016 Nov 14;6:37104. doi: 10.1038/srep37104 (PMC5107903; doi:10.1038/srep37104)
Supplement: Supplementary Information [file srep37104-s1.pdf]

Supplementary information for: **Glycinergic dysfunction in a subpopulation of dorsal horn interneurons in a rat model of neuropathic pain.**

Wendy L Imlach[1], Rebecca F Bhola[1], Sarasa A Mohammadi[1], Macdonald J Christie[1].

[1] The University of Sydney, Discipline of Pharmacology, Sydney, NSW 2006, Australia.

Corresponding author:

Wendy Imlach, email: wendy.imlach@sydney.edu.au

This file includes:

1. Supplementary Figure 1.

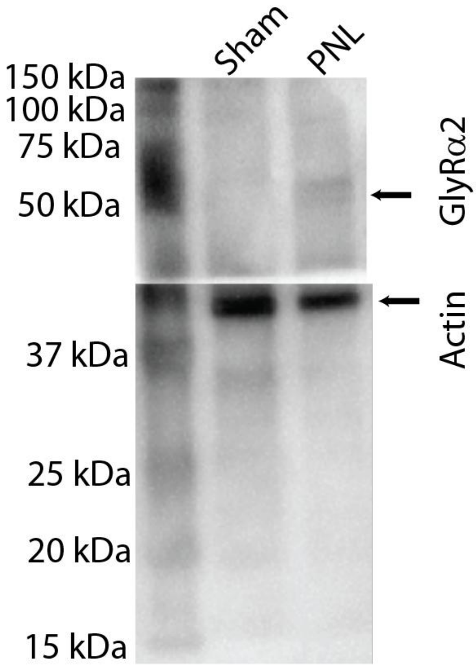

Supplementary Figure 1. Full length western blots of GlyRα2 protein (above) detected with the C-terminal antibody sc-20133[42] (Santa Cruz) and actin loading control (below) detected with an anti-actin antibody (Sigma).
